# Clinical application of flexible fiberoptic bronchoscopy in neonatal respiratory diseases

**DOI:** 10.1186/s13052-024-01755-1

**Published:** 2024-09-20

**Authors:** Xuee Zhuang, Zhiyong Liu, Jingyang Zheng, Jinglin Xu, Dongmei Chen

**Affiliations:** 1 The Graduate School of Fujian Medical University, Fuzhou, China; 2 Department of Respiratory, Quanzhou Maternity and Children’s Hospital, Quanzhou, China; 3 Department of Neonatology, Quanzhou Maternity and Children’s Hospital, Quanzhou, China

**Keywords:** Flexible bronchoscopy, Neonates, Respiratory diseases, Laryngomalacia

## Abstract

**Background:**

Respiratory disease is a predominantly observed problem in neonates. Moreover, the application of flexible bronchoscopy in newborns is gradually increasing. This study aimed to investigate the value of bronchoscopy in neonates respiratory abnormalities and evaluate the safety of bronchoscopy application.

**Methods:**

Clinical data and outcomes of 56 neonates who underwent flexible bronchoscopy were retrospectively analyzed. Correlations among indications for bronchoscopy, findings, and clinical diseases were assessed.

**Results:**

A total of 56 neonates had a minimum weight of 1200 g at the time of bronchoscopy, while the minimum gestational age at birth was 26 + 1 weeks. A total of 22 cases (39.3%) had two or more clinical indications; the five most common indications were respiratory distress in 24 (42.9%), stridor in 22 (39.3%), pulmonary atelectasis in 10 (17.6%), feeding difficulty in 10 (17.6%), and difficult weaning from mechanical ventilation in 6 (10.7%) cases. A total of 13 types of abnormalities were detected in the respiratory tract. The most common abnormalities were laryngomalacia in 29 (59.2%), tracheobroncomalacia in 8 (16.3%), and vocal cord paralysis in 6 (12.2%) cases. Bronchoalveolar lavage was performed in 39 cases. Eight cases were diagnosed by bronchoscopy and then treated with surgery in the Thoracic Surgery/Otolaryngology Department; all of them were cured and discharged from the hospital after surgery. No serious complications, such as pneumothorax or shock, occurred in any of the children, of whom none died.

**Conclusions:**

Flexible bronchoscopy could play an important role in diagnosing and identifying respiratory disorders in neonates and be safely used with few serious complications.

**Supplementary Information:**

The online version contains supplementary material available at 10.1186/s13052-024-01755-1.

## Background

Respiratory diseases are one of the most common diseases in neonatal intensive care unit (NICU) [1], including pulmonary hyaline membrane disease, meconium aspiration pneumonia, infectious pneumonia, and bronchopulmonary dysplasia. Congenital respiratory anomalies include laryngomalacia, vocal cord paralysis, subglottic hemangioma, tracheobroncomalacia, and tracheobronchial stenosis. Their clinical manifestations are nonspecific and may include recurrent stridor, hoarseness, respiratory distress, and recurrent lung infections. Moreover, feeding difficulties or sleep disorders in some newborns may be caused by malformations of the upper and lower airways [2, 3]. Some airways defects are part of malformation syndromes/genetic diseases, and this is also what we should pay attention to in clinical practice [4–6].Diagnosing airway malformations based on traditional examinations, including chest radiography, chest computed tomography (CT), and pulmonary ultrasound is difficult [7].

In recent years, with the technological progress of NICUs and the achievements of neonatal nursing, flexible bronchoscopy (FB) has been gradually popularized in neonates [8, 9]; an increasing percentage of airway developmental abnormalities have been detected in the NICU [10]. FB can enter the lung segments and subsegmental bronchi to examine small airway lesions, and the airways can be dynamically and clearly observed to reduce leakage and misdiagnosis [11]. Meanwhile FB can be used for performing treatments, including alveolar lavage, foreign body removal, local drug administration therapy, balloon dilatation airway angioplasty, endotracheal stenting, and laser therapy. Patients with intra-airway lesions that previously required surgical treatment can be given a chance to be cured by FB [12].

In China, little epidemiological data exist on using FB in neonatal intensive care units to diagnose and treat suspected airway abnormalities. In this study, we retrospectively analyzed the data of neonates who underwent FB during a four-year period in a tertiary NICU in a southern Chinese city, and aimed to investigate the value of FB in neonates with congenital respiratory anomalies and evaluate its operational safety.

## Materials and methods

### Study participants

The study population comprised 56 neonates admitted to the NICU in Quanzhou Maternal and Children’s Hospital China, between May 2018 and April 2022, with suspected respiratory abnormalities and who underwent FB. The inclusion criteria were based on the Pediatric Bronchoscopy Guidelines, specifically for neonates with suspected structural malformations or functional abnormalities of the airways [13].

Clinical data such as sex; mode of delivery; gestational age; birth weight; weight at bronchoscopy; time of bronchoscopy; mechanical ventilation during hospitalization; heart rate; respiration; percutaneous oxygen saturation; and body temperature monitored during and after bronchoscopy were retrospectively collected from the hospital’s electronic medical record system. Clinical indications and results of bronchoscopy were also retrospectively collected.

All parents of the neonates agreed to participate and signed an informed consent form before FB or treatment. This study was approved by the Ethics Committee of Quanzhou Maternal and Children’s Hospital, China (Ethics No. 9, 2022) for the collection and organization of the previous data.

### Flexible bronchoscopy operation

FB was performed using the BF-XP290 (2.8 mm outer diameter, 1.2 mm inner diameter with a 1.2 mm working orifice) by OLYMPLUS, Japan. The indications for bronchoscopy have been strictly defined and contraindications excluded. Routine blood analysis, coagulation function, electrocardiogram, and other examinations have been performed before operation. Moreover, indwelling catheters allowing venous access have been placed. To avoid vomiting during the operation, 3 h of preoperative fasting and intravenous fluid support were administered. The operation was performed on a NICU radiation bed equipped with monitoring and resuscitation equipment. Bronchoscopy was performed by two experienced respiratory endoscopists, one NICU physician, and one specialized nurse.

All neonates were sedated intravenously with midazolam (0.1–0.3 mg/kg); patients with suspected laryngeal abnormalities were sedated after upper airway exploration. Based on sedation, 4 mg/kg of lidocaine (2%) was used for topical airway anesthesia by the “spray-as-you-go” technique via the bronchoscope. The bronchoscope was entered the nasal cavity to observe the nasal cavity for bleeding, secretion, and neoplasms. Entering the pharynx, we observed whether there was any posterior fall of the tongue root, abnormal activity, or deformity of the epiglottis, and whether the vocal folds were symmetrical with normal mobility. When the vocal folds were open, the lens was advanced into the trachea to observe the presence of subglottic stenosis with or without exudation, neoplasm, stenosis, or tenderness. Oxygen was administered throughout the operation.

In cases of intraoperative mucosal bleeding, 1 ml of diluted epinephrine (1:10,000) was injected locally through the working orifice of the bronchoscope to stop bleeding. In neonates suspected of esophageal-tracheal fistula, intragastric injection of diluted methylene blue was performed simultaneously with bronchoscopy. Bronchoalveolar lavage (BAL) should be performed if there were endobronchial inflammation or sputum blockage. In that case, the bronchoscope was embedded in the diseased lobe, and in a section of the bronchus saline solution (1–2 ml/kg) was injected through the working orifice, alveolar lavage was performed and then sucked out. Thereafter, the recovered alveolar lavage fluid was sent for culture, biochemistry, and liquid-based examination according to the neonatal condition.

During the entire operation, transcutaneous oxygen saturation, heart rate, and respiration were monitored; oxygen was administered via a nasal catheter, and the oxygen tube was inserted into the posterior nasal passage at a flow rate of approximately 1–3 L/min. If the patient was mechanically ventilated and could tolerate extubation, the endotracheal tube was removed before bronchoscopy to facilitate examination of the airway; otherwise, a three-way tube was directly connected to increase the oxygen concentration to 100% and accessed through the tracheal intubation channel. If the neonates developed cyanosis or experienced decreased heart rate and transcutaneous oxygen saturation during the operation, the operation was suspended, balloon pressurized oxygen was administered, or ventilator-assisted ventilation was performed after withdrawing the bronchoscope. The operation was continued after observing increased transcutaneous oxygen saturation and heart rate.

### Statistical analysis

Statistical analyses were performed using IBM SPSS Statistics for Windows, version 24.0. Descriptive analysis was used, and data conforming to normally distributed measures were expressed as mean ± standard deviation(M ± SD). Non-normally distributed measures were expressed as median (interquartile spacing) [M (P25, P75)]. Categorical information was described using frequencies and percentages.

## Results

### Clinical data

A total of 56 neonates with FB were included, comprising 35 male and 21 female patients; 42 full-term and 14 preterm infants; 30 vaginal births and 26 cesarean deliveries. Birth gestational age was 37.4 ± 4.0 weeks; birth weight was 2812.2 ± 536.6 g. The median weight at the time of bronchoscopy was 3246.79 ± 673.6 g, and the median age 8 days, respectively. The minimum birth weight was 650 g; the minimum weight at the time of bronchoscopy was 1200 g. The minimum gestational age at birth was 26^+ 1^ weeks; the minimum postmenstrual age at the time of bronchoscopy was 34^+ 1^ weeks. A total of 36 patients underwent invasive mechanical ventilation during hospitalization. The patient characteristics are presented in Table 1.

**Table 1:** General characteristics of the patients(n:56)

Male, *n*(%)	35(62.5%)
Female, n(%)	21(37.5%)
Premature infants, n(%)	14(25.0%)
Gestational age at birth(M ± SD), weeks	37.4 ± 4.0
Birth weight(M ± SD), g	2812.2 ± 536.6
Weight at the time of FB(M ± SD), g	3246.76 ± 673.6
Median age at bronchoscopy(IQR), days	8(1–15)

Abbreviations: M:mean, SD: standard deviation FB: flexible bronchoscopy

IQR: interquartile range

**Fig. 1:**
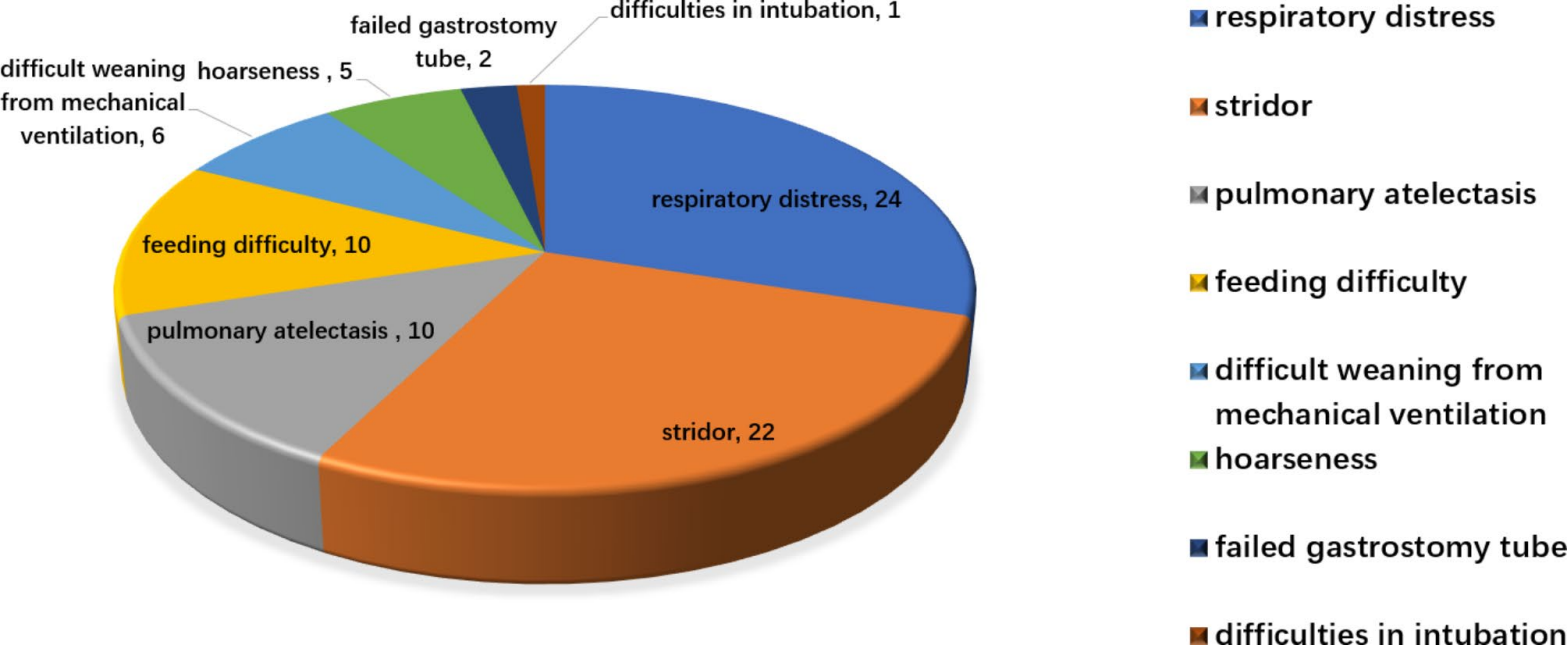
Clinical indication of bronchoscopy in 56 neonates with suspected respiratory malformation

The clinical indications for 56 neonates undergoing FB were: respiratory distress, 24 (42.9%); stridor, 22(39.3%); pulmonary atelectasis, 10(17.6%); feeding difficulty, 10 (17.6%); weaning difficulties from mechanical ventilation, 6(10.7%); hoarseness, 5(8.9%); failure to place a gastrostomy tube, 2 (3.6%); and difficulties in intubation ,1(1.8%) cases(Fig. 1). A total of 34 (60.7%), 20 (35.7%), and 2 (3.6%) cases had a single, two, and three clinical indications, respectively.

### Findings of flexible bronchoscopy

**Fig. 2:**
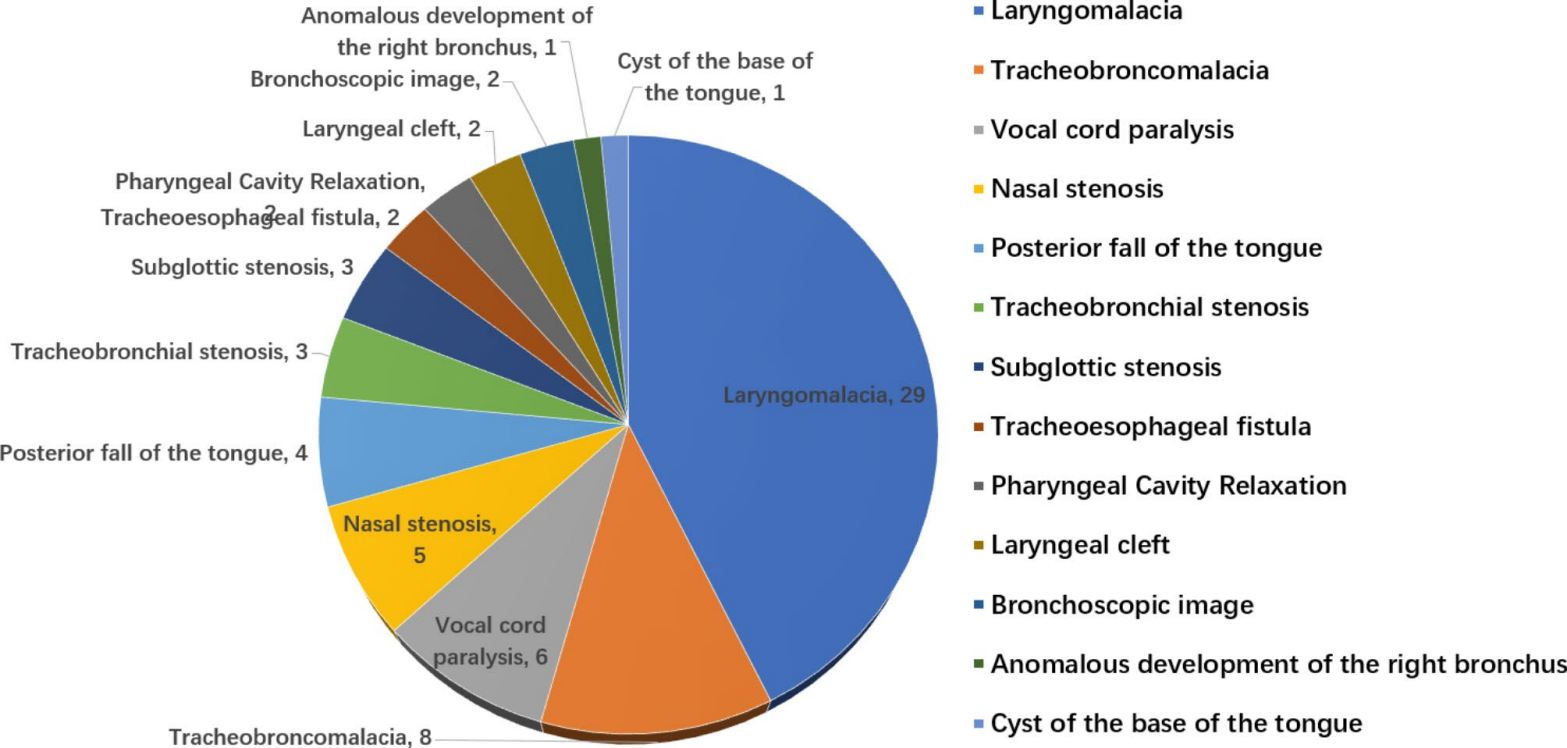
Composition of respiratory tract abnormalities detected by flexible bronchoscopy in 56 neonates

**Fig. 3:**
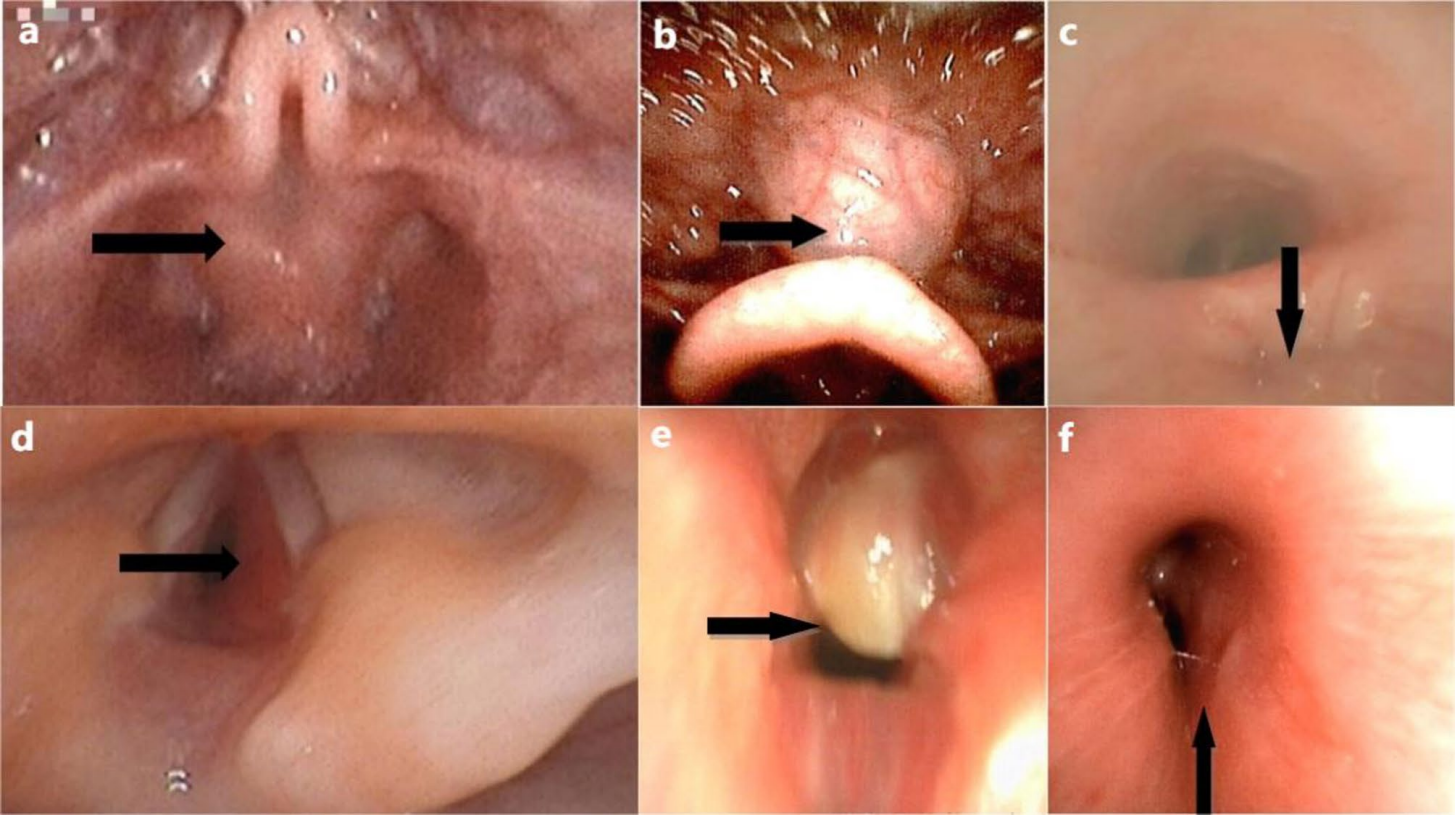
Different types of respiratory abnormalities detected by flexible bronchoscopy. (**a**) Laryngomalacia. (**b**) Tongue base cyst. (**c**) Esophagotracheal fistula. (**d**) Subglottic hemangioma. (**e**) Glottic occupation. (**f**) Left main bronchial stenosis

Of the 56 FBs in this study, 7 cases suggested simple inflammation of the endobronchial lining. Airway abnormalities were detected in 49 patients (87.5%); of these, 28(57.1%) had a single abnormality and 21 (42.9%) had two or more abnormalities. A total of 13 types of abnormalities were detected in the 49 cases, including 7 abnormalities in the upper respiratory tract and 6 in the lower respiratory tract. The five most common types were laryngomalacia in 29(59.2%), tracheobroncomalacia in 8(16.3%), vocal cord paralysis in 6(12.2%), nasal stenosis in 5(10.2%), posterior fall of the tongue in 4(8.2%) cases (Fig. 2). Typical neonatal respiratory abnormalities are shown in Fig. 3.

Laryngomalacia was the most common endoscopic manifestation in all indications except for failure of gastric tube insertion (Table 2).

**Table 2:** Correlation between bronchoscopy clinical indication and bronchoscopy finding in 56 neonates

clinical indication	Laryngomalacia	Tracheobroncomalacia	Vocal cord paralysis	Nasal stenosis	Subglottic stenosis	Tracheobronchial stenosis	Posterior fall of the tongue	Cyst of the base of the tongue	Laryngeal cleft	Anomalous development of the right bronchus	Bronchoscopic image	Tracheoesophageal fistula	Pharyngeal Cavity Relaxation
Respiratory distress	13	4	1	1	1	2	2	-	1	1	2	-	2
Stridor	17	1	5	2	3	-	-	1	1	-	-	-	1
Pulmonary atelectasis	5	5	1	-	-	-	-	-	-	1	-	-	1
Feeding difficulty	5	-	3	-	-	-	2	-	1	-	-	-	1
Difficult weaning from mechanical ventilation	1	-	1	1	1	-	-	-	-	-	-	-	-
Hoarseness	3	-	1	1	-	-	-	-	-	-	-	-	-
Failed gastrostomy tube	-	2	-	-	-	-	-	-	-	-	-	2	-
Difficulties in intubation	1	-	-	-	-	1	-	-	-	-	-	-	-

-: no case

### Treatment and interventions during or after flexible bronchoscopy

Among the 56 neonates, 7 cases of simple endobronchial infection and 32 cases of combined endobronchial infection underwent BAL. BAL fluid was aspirated and sent for pathogen culture in 18 cases and revealed positive results in 7 cases (41.2%); according to drug sensitivity results, the treatment was adjusted to sensitive antibiotics, after which the condition improved significantly. Surgical treatment was performed in the Department of Otorhinolaryngology after establishing diagnosis in 6 cases; their symptoms improved, and they were discharged from the hospital. Two cases of esophagotracheal fistula were operated in the Thoracic Surgery Department, and there cured. They were discharged after undergoing thoracoscopic exploration + esophageal-tracheotracheal fistula ligation + open thoracic esophageal fistula ligation + closed chest drainage. Table 3 presents the specific treatments and interventions.

**Table 3:** Treatment and intervention during or after bronchoscopy in 56 neonates

Intervention or surgery	case	Results
bronchoalveolar lavage	39	Clinical benefit
endoscopic low-temperature plasma-assisted partial ablation of the lower left middle turbinate and inferior turbinate	1	Healed and discharged
plasma-resection of laryngeal mass under the support of laryngoscope	1	Healed and discharged
endoscopic plasma-assisted bilateral posterior nostriplasty + left nasal vestibular mass resection + nasal septum cyst fenestration and drainage	1	Healed and discharged
external mobilization of the left vocal cord under the support of laryngoscope + cricolaryngotomy + supragloplasty	1	Healed and discharged
supragloplasty under the support of laryngoscope	1	Healed and discharged
under the support of laryngoscope iso-dissociation-assisted ablation of the lower root of the tongue + supragloplasty	1	Healed and discharged
thoracoscopic exploration + esophagotracheal fistula ligation + esophagotracheal reconstruction + thoracic closed drainage	2	Healed and discharged

### Complications

Among the 56 cases of flexible bronchoscopy, 10 cases (17.6%) had a transient decrease in transcutaneous oxygen saturation to approximately 80%; after management such as suspending bronchoscopy and back tapping and oxygen, the conditions improved rapidly and the cases were able to tolerate until the operation was completed. In addition, eight cases (14.3%) had bronchial mucous membrane injuries with slight bleeding, which were treated by injecting 1 ml of diluted adrenaline (1:10,000) into the working channel of the bronchoscope to locally stop the bleeding, and local hemostasis was achieved in all patients. No serious bleeding complications were observed. Pneumothorax, shock, or death did not occur in any of the children.

## Discussion

Respiratory disorders are the main reason of newborns admissions to the intensive care unit [14]. In the past, neonates with congenital respiratory abnormalities may not have been accurately diagnosed on time due to the difficulty in obtaining suitable FB equipment. With continuous improvements in respiratory endoscopy techniques, different bronchoscopic equipments are available for neonates and adults. Bronchoscopy has both diagnostic and therapeutic value in neonates [10]. The purpose of the examination is mainly to diagnose and assess airway function and the degree of airway deformity, including working orifices for oxygenation, negative pressure suction, alveolar lavage, and pathologic biopsy. Overseas, it was reported in the 1980s that it could be used to examine and treat preterm infants weighing 600 g [15]. In our study, the minimum postmenstrual age of neonates undergoing bronchoscopy was 34^+ 1^ weeks, and the minimum weight was 1200 g, confirming the need for bronchoscopy in neonates of smaller gestational age and weight.

Common indications for bronchoscopy in neonates include unexplained cyanosis, respiratory distress, and difficulty in weaning from mechanical ventilation [16, 17]. In this report, the top two clinical indications were respiratory distress (42.9%) and stridor (39.3%), consistent with the findings of many reports [18, 19]. Several analyses have confirmed that respiratory distress and stridor are common clinical manifestations of developmental malformations of the respiratory airways in neonates. Wang et al. retrospectively analyzed the data of 175 infants and children with unexplained dyspnea, suggesting that its etiology varies in different age groups, with respiratory malformations predominating up to 6 months of age and the proportion of respiratory malformations tending to decrease with increasing age [18]. A study in Saudi Arabia showed that laryngomalacia in 217 children with stridor was detected at a much higher rate in the younger age group (< 1 year) than in the older age group (> 1 year) (83% vs. 43%, *P* < 0.001) [19]. Therefore, in clinical practice, neonates with unexplained respiratory distress and stridor, who are suspected of having an airway abnormality that cannot be clarified by imaging examination, should undergo bronchoscopy to evaluate the airway and help identify the etiology at an early age.

In a review of 27 pediatric analyses, Field-Ridley et al. reported that FB helped diagnose 82% of patients [20]. This rate was even higher in patients with suspected airway disease and ventilator-dependent patients [21]. Ke et al. reported that 147 neonates with FB showed at least one positive finding in 95.92% of cases [22]. FB can be increasingly utilized in clinical practice, providing new ideas and directions of diagnosing and treating complex diseases [23]. The high diagnostic contribution of FB was emphasized by the 87.5% detection rate of airway abnormalities in the 56 neonates of our study. Overall, roughly 64–91% of neonates undergoing bronchoscopy in the NICU were found to have one congenital defect [24–26]. The high incidence of abnormal findings may be linked with the stricter indications adopted by physicians for early bronchoscopy in neonates.

Both detection rate and variety of respiratory anomalies are high among newborns compared with elder children. Not only is the detection rate of respiratory malformations high among newborns, but also the variety of respiratory anomalies is high compared with the older children [27]. A total of 13 types of respiratory tract abnormalities were detected in this study, with laryngomalacia topping the list of upper respiratory tract abnormalities, followed by abnormal vocal cord movement, while tracheobronchial malacia is the most frequently observed among the lower respiratory tract anomalies, followed by tracheal stenosis. Congenital laryngomalacia was previously thought to result from the development of immature laryngeal cartilage.

The prevalence of preterm infants in our study (7/14, 50%) did not increase compared with full-term infants (22/42, 52.3%), which is in agreement with the findings of Schroeder et al. [28]. A retrospective analysis of 162 neonates bronchoscopy findings by Billings et al. showed that the top three developmental respiratory tract anomalies were laryngomalacia (39.0%), subglottic stenosis (31.9%), and tracheomalacia (25.8%) [29].

FB is considered an excellent diagnostic tool for laryngomalacia [30, 31]. In the present study, laryngomalacia was the predominant presenting finding under different clinical indications for bronchoscopy, except for indwelling gastric tube failure. More than 50% of children with laryngomalacia present with secondary airway pathologies, with subglottic stenosis and tracheomalacia being the most common [32]. In our study of the 29 patients identified, in 17 (58.6%) it was associated with other anomalies. In addition to laryngomalacia detected by bronchoscopy in neonates, vigilance should be exercised for the comorbidity of other airway anomalies to avoid missed diagnoses.

FB combined with BAL is an important method for diagnosing and treating severe pneumonia [33, 34]. A total of 39 neonates underwent BAL and achieved good therapeutic results; 6 other neonates underwent pharyngeal surgery after bronchoscopy, and two newborns underwent thoracoscopic surgery. More reports have confirmed that bronchial intervention techniques are increasingly utilized in clinical practice, providing new ideas and directions for diagnosing and treating complex diseases in neonates [35–37].

Many analyses have reported the safe use of bronchoscopy in neonates [22, 29, 34, 37]. Among them, hypoxemia and mucosal bleeding are the common complications. In this study, the most common complications were hypoxemia (17.6%) and airway mucosal hemorrhage (14.3%). All patients recovered uneventfully after symptomatic management. No serious complications, such as pneumothorax, hemorrhage, or shock occurred in any of the children. This shows that bronchoscopy is safe and reliable in neonates, and even in preterm and very preterm infants, with adequate preparation and skillful operation during examination.

This study has some limitations. First, this was a single-center retrospective study with a small sample size, which is prone to bias and requires a larger sample size for further confirmation. Second, except for alveolar lavage, the rest of the operations such as laser, balloon dilatation, and stent placement have not been performed in this study. In the future, we can explore these techniques to provide more clinical evidence for applying neonatal bronchoscopic interventional techniques.

## Conclusions

The clinical symptoms of neonatal respiratory disorders appear early, including respiratory distress, stridor, and feeding difficulties. However, there is no specificity in the clinical manifestations of different types of respiratory disorders. FB plays an important role in diagnosing and identifying respiratory disorders in neonates and can be safely used with few serious complications.

## Electronic supplementary material

Below is the link to the electronic supplementary material.

Supplementary Material 1

## Data Availability

The datasets used and analyzed in the current study are available from the corresponding author upon reasonable request.

## Abbreviations

FB: Flexible bronchoscopy
NICU: Neonatal intensive care unit
BAL: Bronchoalveolar lavage
CT: Computed tomography
