# Supplementary material for: Clinical application of flexible fiberoptic bronchoscopy in neonatal respiratory diseases
Source: Ital J Pediatr. 2024 Sep 20;50:190. doi: 10.1186/s13052-024-01755-1 (PMC11415989; doi:10.1186/s13052-024-01755-1)
Supplement: Supplementary file 1 — Supplementary Material 1 [file 13052_2024_1755_MOESM1_ESM.docx]

Dear Reviewers:

We thank you so much for your letter and for the comments regarding our manuscript entitled “Clinical application of flexible fiberoptic bronchoscopy in neonatal respiratory diseases”. These comments were all valuable and very helpful for revising and improving our paper. We have studied the comments carefully and have made correction which we hope that they will be met with approval. The main corrections in the paper and the point-by-point response to the comments are as follows:

Our Point-by-point response to the comments and suggestions is listed below:

**Minor revision**

1.line 56, please change with "Some airways defects are part of malformation syndromes/genetic diseases, and this is also what we should pay attention to..." (to avoid repetitions and use the same verb tenses)

**Response:** We thank very much for this careful guidance. Accordingly, change have been made. (line:56-57)

2.Line 117, please remove the repetition "was injected"

**Response:** Frist of all, we apologize for the mistake. We had removed it. (line:117)

3.line 182, please remove the semicolon after "was completed"

**Response:** We thank you for pointing this out. We had removed it. (line:181)

4.line 218, change with "the 95.92% of cases" (instead of patients, to avoid repetitions)

**Response:** Thank you very much for your suggestion. Accordingly, change have been made. (line:218)

5.line 218, the sentence "The prevalence of preterm infants, the incidence of airway malformations was higher," must be removed, as it is a misunderstanding of a previous suggestion

**Response:** We thank very much for the careful guidance. We had removed it.

6.line 235, the sentence "the prevalence of preterm infans in our study..." should me moved to a new line

**Response:** We thank you for the insightful suggestion. Therefore, we corrected it. (line:235)

7.line 254, change with "Many analyses have reported"

**Response:** We thank you for the advice. Through the advice, the expressions are indeed better. (line:254)

We have tried our best to improve the manuscript and made some changes to the manuscript. We appreciate your comments and suggestions, and hope that the correction will be met with approval.

Thank you and best regards.

Yours sincerely,

Dongmei,Chen
